# Diagnostic impact of whole exome sequencing in neurometabolic disorders in Syrian children: a single center experience

**DOI:** 10.1186/s13023-025-03732-1

**Published:** 2025-05-07

**Authors:** Rawan Al Khudari, Sameer Baqla, Diana Al Asmar

**Affiliations:** 1 Department of Pediatrics, Children’s University Hospital, Faculty of Medicine, Damascus University, Damascus, Syria; 2 Division of Neurology, Department of Pediatrics, Children’s University Hospital, Faculty of Medicine, Damascus University, Damascus, Syria; 3 Division of Inborn Errors of Metabolism, Department of Pediatrics, Children’s University Hospital, Faculty of Medicine, Damascus University, Damascus, Syria

**Keywords:** Whole exome sequencing, Neurometabolic disorder, Genetic diagnostics, Novel mutations

## Abstract

**Background:**

Childhood neurometabolic disorders encompass a range of heterogeneous conditions often presenting with atypical or overlapping symptoms, making accurate diagnosis challenging, time-consuming, and costly. Whole exome sequencing (WES) has recently become a valuable diagnostic tool for suspected genetic or idiopathic neurometabolic disorders. This study evaluates the diagnostic utility of WES in Syrian patients with neurological and metabolic disorders, marking the first report of WES outcomes in this context.

**Results:**

Among 54 patients, 42 (78%) were from consanguineous families, of whom 38 (90%) had positive WES results. WES identified pathogenic or likely pathogenic variants in 28 patients (52%) and discovered 14 novel mutations. Seventeen patients (31%) had variants of uncertain significance (VUS) aligning with their clinical presentation, and nine (17%) had negative results. WES provided clinically relevant information for 45 patients (83%), with a definitive diagnosis in 28 (52%). Additionally, WES led to diagnostic changes in 45 cases (83%) and treatment alterations in 40 cases (74%).

**Conclusion:**

Our findings demonstrate the high diagnostic yield of WES and its substantial impact on clinical outcomes. WES has facilitated changes in diagnosis, treatment adjustments, prognostic modifications, and preventive measures, supporting its utility in undiagnosed neurometabolic diseases. This study advocates for WES in pediatric neurometabolic cases, particularly where consanguinity is present.

## Introduction:

Childhood neurometabolic disorders are inherited conditions that, although individually rare, collectively impose a significant burden. These disorders often present with atypical or overlapping clinical features, making their diagnosis challenging, time-consuming, and costly [1, 2].

The term"exome"refers to the complete set of exons in the human genome, which are the approximately 180,000 genomic sequences that are transcribed and retained in mature RNA. Although the exome constitutes only 3% of the human genome, the exome is linked to nearly 85% of clinically significant genetic disorders [3]. Consequently, whole exome sequencing (WES) has emerged as a highly effective and efficient method for identifying the genetic basis of diseases. WES is particularly valuable in detecting rare mutations in autosomal recessive disorders, especially in populations with a high prevalence of consanguinity [4].

In recent years, WES has emerged as a pivotal diagnostic tool for genetic and idiopathic neurometabolic disorders. It is cost-effective and offers a more expedient pathway to diagnosis. Accurate diagnosis is critical for providing appropriate genetic counseling to families and preventing the recurrence of similar conditions [1].

In the Middle East, particularly in Syria, consanguinity is prevalent, which may contribute to a higher incidence of neurometabolic disorders. However, few studies in the Middle East have examined the outcomes of WES in children with these conditions [1]. The purpose of this study is to evaluate the diagnostic utility of WES in patients with neurological and metabolic disorders. The advent of next-generation sequencing has facilitated the rapid identification of rare and novel genetic disorders, significantly impacted medical management and reducing costs. This study is the first to describe the diagnostic rate, outcomes, advantages, and limitations of WES in Syria.

## Method

A retrospective analysis was conducted on 54 children with undiagnosed neurometabolic conditions between January 2020 and March 2024. The neurological disorders observed ranged from developmental delay and hypotonia to seizures, ataxia, white matter changes, intellectual delay, encephalopathy and metabolic abnormalities. The study was conducted at the Children's University Hospital and the metabolic and neurology clinic in Damascus, Syria.

The informed consent of each patient was obtained from their parents, and ethical approval was attained from the local Ethics Committee of Damascus University.

The medical records of the patients were collated by means of both paper and electronic documentation, with databases subsequently created from these records. The characteristics of the patients are presented in Table 1.

**Table 1:** The patient demographic characteristics

Characteristics	Total	Positive n(%)		Negative wes n(%)
		Positive WES (pathogenic, likely pathogenic)	VUS in WES (reclassified as having clinically significant variants after reevaluation of phenotype)	
Total number (n)	N = 54	N = 28 (52%)	N = 17 (31%)	N = 9 (17%)
Age (mean)	4 years (4 months-14 years)	4 years (4 months-14 years)		3 years (1–6 years)
Gender (M:F)	27/27	22:23		5:4
Consanguineous families	N = 42 (78%)	38 (90%)		4 (10%)
POSITIVE family history	N = 28 (52%)	24 (86%)		4 (14%)

The demographic data of the patients was reviewed and documented, together with details of their history of consanguinity, the clinical examination and the results of the neuroimaging. All patients underwent whole-exome sequencing (WES). The whole exome sequencing was conducted at an accredited laboratory, Centogene in Rostock, Germany. The variant classifications were based on the American College of Medical Genetics and Genomics (ACMG) guidelines [5] and were divided into five classes.

Class I is pathogenic, class II is likely pathogenic, class III is variant of uncertain significance (VUS), class IV is likely benign and class V is benign.

In order to evaluate the utility of WES in terms of its impact on diagnosis and clinical decision-making, a 5-point scale was devised. Two points would be awarded for a change in the patient's diagnosis, one point for a change in the treatment plan, one point for a change in preventive measures and one point for a change in prognosis.

Parental satisfaction was assessed using a simple yes/no response. We acknowledge that cultural factors, such as deference to medical professionals, may influence parental satisfaction responses. To minimize this bias, parents were encouraged to provide candid feedback in a neutral setting. Parental satisfaction was assessed using a simple yes/no response. We acknowledge that cultural factors, such as deference to medical professionals, may influence parental satisfaction responses. To minimize this bias, parents were encouraged to provide candid feedback in a neutral setting.

## Result

A total of 54 patients (50% female) were included in the study. The mean age of presentation was four years, with a range of four months to 14 years.

Of the 54 patients, 42 (78%) were from consanguineous families, with 38 of these 42 (90%) exhibiting a positive WES result. A neurological or metabolic disorder family history was reported in another sibling or relative in 28 patients. (Table 1).

The patients presented with a range of neurological manifestations, including seizures (in 21 patients, representing 39% of the cohort), motor delay (in 11 patients, or 20% of the cohort), regression of developmental milestones (in 36 patients, or 67% of the cohort), hypotonia (in 15 patients, or 28% of the cohort), and speech and language delay (in 22 patients, or 41% of the cohort). Eleven patients (20%) presented with abnormal gait. Additionally, three patients (5.5%) exhibited dysarthria. Additionally, three patients (5.5%) exhibited spasticity, while 20 patients (37%) demonstrated abnormalities on magnetic resonance imaging (MRI) of the brain.

The median duration between the onset of symptoms and the genetic diagnosis via whole exome sequencing (WES) was four months (one to 36 months).

### WES Outcomes

Whole exome sequencing (WES) results were positive in 28 patients, with pathogenic or likely pathogenic variants identified in 52% of cases. Seventeen patients (31%) had variants of uncertain significance (VUS) that were consistent with their clinical presentation, while the remaining nine patients (17%) had negative results. Four patients were diagnosed with two distinct genetic disorders: two had dual autosomal recessive conditions, and two had an autosomal recessive disorder along with a mitochondrial gene disorder.

**Fig. 1:**
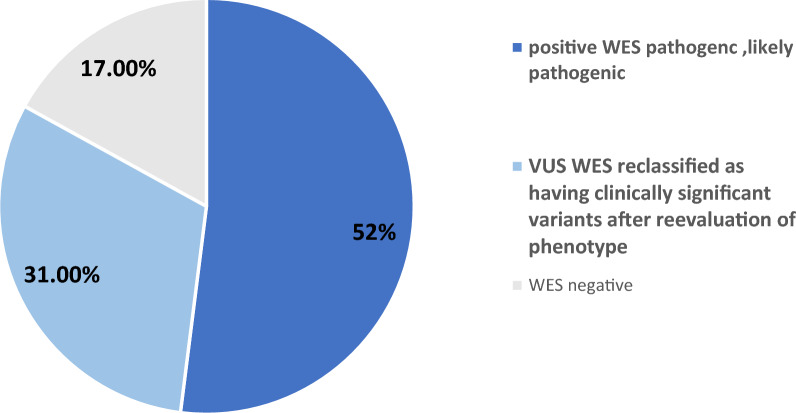
Diagnostic rate of WES: positive and negative WES

The WES-positive results were categorized into pathogenic, likely pathogenic variants, and VUS. Pathogenic and likely pathogenic mutations were found in 22 and 6 patients, respectively. These mutations were consistent with the patients'phenotypes, confirming the clinical diagnosis. In the VUS group, 17 patients were primarily reclassified as having clinically significant variants, as their genotypes matched their phenotypes (Fig. 1).

**Fig. 2:**
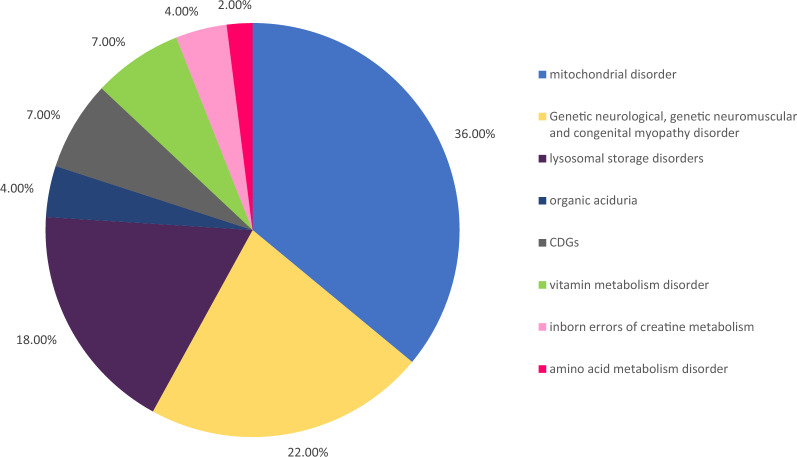
Prevalence of the diseases diagnosed

As a result, WES provided clinically useful information in 45 patients (83%) and a definitive diagnosis in 28 patients (52%). A WES diagnosis of a neurometabolic disease was made in 16 patients (36%) with a mitochondrial disorder (Fig. 2), 8 patients (18%) with a lysosomal disorder, and 10 patients (22%) with either a genetic neurological disorder, a genetic neuromuscular disorder, or a congenital myopathy disorder. Additionally, 2 patients (4%) had organic aciduria, 3 patients (7%) had congenital disorders of glycosylation (CDGs), 3 patients (7%) had a vitamin metabolism disorder, 2 patients (4%) had inborn errors of creatine metabolism, and 1 patient (2%) had an amino acid metabolism disorder (Fig. 2, Table 2). Of the 54 patients, 44 (81%) had autosomal recessive conditions, one (2%) had an autosomal dominant condition, and 4 (7%) had mitochondrial genetic disorders (Tables 3 and 4).

**Table 2:** Disease prevalence

	N(%)	Comments
Mitochondrial disorder	N = 16 (36%)	Leigh syndrome n = 3 primary coenzyme Q10 deficiency n = 1 molybdenum cofactor deficiency of complementation group A n = 1 Disorders of Pyruvate Metabolism n = 2 mitochondrial complex deficiency n = 6 MT-ATP6 gene-associated disorder n = 1 MT-TS1 gene-associated mitochondrial disorders n = 1 mitochondrial DNA depletion syndrome n = 1
Genetic neurological, genetic neuromuscular and congenital myopathy disorder	N = 10 (22%)	Developmental and epileptic encephalopathy type 48. n = 1 Cerebroretinal microangiopathy with calcifications and cystic n = 1 epilepsy early onest,4, vitamin b6-dependent n = 1 Galloway-Mowat syndrome n = 1 Hypomyelinatingleukodystrophy n = 1 Spastic paraplegia n = 1 IGHMBP2-related disease. N = 1 autosomal recessive minicore myopathy with external ophthalmoplegia. n = 1 spectrum of TPM2 gene n = 1 Vici syndrome n = 1
Lysosomal disorders	N = 8 (18%)	Sandhoff syndrome n = 1 Tay Sachs n = 4 Niemann-Pick disease C n = 1 mucolipidosis II alpha/beta n = 1 mucopolysaccharidosis type IIIA (Sanfilippo A) n = 1
Organic aciduria	N = 2 (4%)	Glutaric aciduria n = 2
CDGs	N = 3 (7%)	Congenital disorders of glycosylation (CDGs) n = 3
Vitamin metabolism disorder	N = 3 (7%)	Thiamine metabolism dysfunction syndrome n = 1 pyridoxamine 5-prime-phosphate oxidase deficiency n = 1 methylmalonic aciduria with homocystinuria n = 1
Inborn errors of creatine metabolism	N = 2 (4%)	Cerebral creatine deficiency syndrome type 2 n = 2
Amino acid metabolism disorder	N = 1 (2%)	BH4-deficient hyperphenylalaninemia n = 1

**Table 3:** Pathogenic and likely pathogenic variant in Autosomal Dominant Inheritance and Autosomal recessive Inheritance

Sex-age	Consan Guinity	Family history	Genes	Variant	Diagnosis	Comments
Autosomal Dominant Inheritance						
M-3 Y	NO	YES	TPM2	heterozygous pathogenic: *NM_001301226.1:c.415_417 del*	Disorder with the phenotypic spectrum of TPM2 gene	Hypotonia
Autosomal recessive Inheritance						
M-6Y	NO	NO	HEXA	NM_001318825.1:c.1544 G > A	GM2 gangliosidosis type 1 (Tay Sachs)	Motor and language delays -dysarthria
F-8 M	YES	YES	ALDH7 A1	NM_001182.3:c.1597 del	Epilepsy early onest,4, vitamin b6-dependent	Hypotonia-seizures
F-13Y	YES	NO	TTC19	**NM_017775.3:c.779_780 del**	Mitochondrial complex III deficiency nuclear type 2	Motor and language delays
F-4Y	YES	YES	SURF1	NM_003172.3: c.845_846 del	Mitochondrial complex IV deficiency nuclear type 1	regression of milestones- Motor delay
F-8Y	YES	NO	HEXA	**NM_001318825.1: c.1544G > A**	GM2 gangliosidosis (Tay Sachs)	Motor and language delays
F-8Y	Yes	No	SGSH	**Likely pathogenic: NM_000199.3: c.1093 C > T**	Mucopolysaccharidosis type IIIA (Sanfilippo A)	Motor and language delays
F-7Y	Yes	Yes	HEXA	NM_001318825.1:c.566G > A	GM2 gangliosidosis (Tay Sachs)	Motor and language delays—seizures
M-4Y	NO	NO	MOCS1	NM_001075098.3: c.722 del	Molybdenum cofactor deficiency of complementation group A	Regression of milestones- Motor delay- hypotonia-seizures
M-14Y	YES	NO	GCDH	NM_000159.2: c.532G > A	Glutaric aciduria type 1	Motor delay- gait impairment- Tandem Mass Spectrometry (TMS) normal
F-6Y	YES	NO	QDPR	NM_000320.2:c.661 C > T	BH4-deficient hyperphenylalaninemia type C	Regression of milestones- Motor delay- thrombosis -seizures- Phenylketonuria
M-3Y	YES	NO	MMACHC	NM_015506.2:c.271 dup	Methylmalonic aciduria and homocystinuria type cblC (MAHCC)	Seizures- hypotonia
F-1Y	NO	NO	EPG5	NM_020964.2:c.895 C > T	Vici syndrome	Abnormality of retinal pigmentation; Agenesis of corpus callosum; Albinism; Delayed social development; Dysphagia; Growth delay; Motor delay; Seizure
M-10Y	YES	NO	AP3B2	**Likely pathogenic: NM_001278512.1: c.3235_3238 del**	Developmental and epileptic encephalopathy type 48	Abnormal circulating glutamine concentration; Delayed language Growth delay; Infantile onset; Seizure;
F-1Y	NO	NO	PET100	NM_001171155.1:c.3G > C	Mitochondrial complex IV deficiency nuclear type 12	Seizures, hypotonia, Cerebral atrophy; Hyperammonemia; acidosis; Leukoencephalopathy;
M-2Y	YES	NO	SLC19 A3	NM_025243.3:c.68G > T	Thiamine metabolism dysfunction syndrome type 2	Motor and language delays, Spasticity
M-7Y	NO	YES	TTC19	**M_017775.4:c.779_780 del**	Mitochondrial complex III deficiency nuclear type 2	Motor and language delays
F-1Y	YES	YES	SURF1	likely pathogenic NM_003172.3:c.370G > A	Leigh syndrome	Regression of milestones- Motor delay- hypotonia, Abnormal basal ganglia MRI signal intensity; Abnormal caudate nucleus morphology; Abnormal globus pallidus morphology;
F-9Y	YES	NO	IGHMBP2	**Likely pathogenic: NM_002180.2: c.2601_2604 del**	IGHMBP2-related disease. Charcot-Marie-Tooth disease type 2S	Hypotonia
F-4Y	YES	YES	PDHX	NM_003477.2:c.742 C > T	Pyruvate dehydrogenase E3-binding protein deficiency	Motor and language delays
F-3Y	YES	NO	AP4S1	NM_007077.3: c.124 C > T	Spastic paraplegia type 52	Cerebral atrophy; Coarse facial features; Corpus callosum atrophy; Delayed speech and language development
M-1Y	YES	NO	SUCLA2	Likely pathogenic NM_003850.2:c.985 A > G	Autosomal recessive mitochondrial DNA depletion syndrome type 5	Hypotonia-motor delay
M-3Y	YES	YES	HEXB	NM_000521.3:c.1613 + 15_1613 + 18 dup	Sandhoff disease	Hypotonia—motor and languages delay-seizures
M-8Y	YES	YES	HEXA	NM_001318825.1:c.1544G > A	GM2- gangliosidosis type 1 (Tay-Sachs disease)	Dysarthria- motor delay- gait impairment
M-3Y	YES	YES	WDR73	likely pathogenic NM_032856.3:c.287G > A	Galloway-Mowat syndrome type1	Motor delay, Frontotemporal cerebral atrophy

Novel mutations in bold, All mutations were homozygous, except those in Italics which were heterozygous.*TMS* Tandem Mass Spectrometry

**Table 4:** variant of uncertain significance VUS in Autosomal Dominant Inheritance and Autosomal recessive Inheritance

Sex-age	Consan-Guinity	Family history	Genes	Variant	Diagnosis	Comments
Autosomal recessive Inheritance						
M-11Y	YES	NO	COQ8 A	**NM_020247.4: c.1166 T > C**	Primary coenzyme Q10 deficiency type 4	Gait impairment, dysarthria
F-10Y	NO	NO	GCDH	NM_000159.2: c.1045G > A	Glutaric aciduria type 1	Regression of milestones- Motor delay- Spasticity
F-4Y	YES	YES	PC PKLR	VUS: **PC, c.355G > A p.(Gly119 Arg) NM_000920.3:c.355G > A** Pathogenic: PKLR, c.1456 C > T p.(Arg486 Trp)	Pyruvate carboxylase deficiency and Pyruvate kinase deficiency	Regression of milestones- Motor delay-anemia
F-6Y	YES	NO	COX10 BCKDHA	VUS: NM_001303.3:c.727_729 del COX10, c.727_729 del p.(Cys243 del) *Pathogenic: BCKDHA, c.890G* > *A p.(Arg297His)*	Leigh syndrome due to mitochondrial COX4 deficiency and the patient is carrier of the BCKDHA variant maple syrup urine	Motor delay- gait impairment- languages delay- seizures
M-12Y	YES	YES	GAMT	NM_138924.2:c.391 + 5G > C	Cerebral creatine deficiency syndrome type 2	Motor delay- gait impairment- seizures
M-2Y	YES	YES	SLC35 A1	**NM_006416.4:c.508- 6 T > C**	Congenital disorders of glycosylation (CDGs)	Motor and languages delay, Abnormal circulating monocarboxylic acid concentration; Cerebral atrophy,; Elevated circulating 4-hydroxyphenylacetic acid concentration
F-2y	Yes	Yes	SLC35 A1	**NM_006416.4:c.508- 6 T > C**	Congenital disorders of glycosylation (CDGs)	Motor and languages delay, Abnormal circulating monocarboxylic acid concentration; Cerebral atrophy,; Elevated circulating 4-hydroxyphenylacetic acid concentration
M-2Y	YES	YES	DPAGT1	NM_001382.3:c.1195 T > A	Congenital disorder of glycosylation type Ij	regression of milestones- Motor delay-seizures – Leukoencephalopathy- hypotonia
F-3Y	YES	YES	GAMT	**NM_138924.2:c.407 C > G**	Cerebral creatine deficiency syndrome type 2	Abnormal globus pallidus morphology; Brain imaging abnormality; Increased circulating lactate dehydrogenase concentration; Microcephaly; Motor delay; Seizure
F-1Y	YES	YES	NDUFS1 MT-ND3	VUS: NM_001199984.1:c.236 A > G p.Tyr79 Cys the mitochondrial gene MT-ND3 in homoplasmic NC_012920.1:m.10236 A > G ***also see ***Table 5	Mitochondrial complex I deficiency nuclear type 5 AND mitochondrial DNA-associated Leigh syndrome	Motor delay; Seizure
M-4Y	YES	YES	MBOAT7 MT-TS1	VUS: MBOAT7, c.121 del p.(Leu41Serfs*68) heteroplasmic pathogenic: MT-TS1, c.26 dupC ***also see ***Table 5	Mental retardation type 57 AND mitochondrial MT-TS1 gene-associated mitochondrial disorders	Global developmental delay, languages delay Abnormal vitamin B12 level; Frontal cortical atrophy;
F-3Y	YES	YES	RYR1	NM_000540.2:c.2654G > A	Minicore myopathy with external ophthalmoplegia	Global developmental delay, hypotonia, seizures
M-2Y	YES	YES	CTC1 EIF2B5	**NM_025099.5: c.153G > T** **NM_003907.2: c.1105G > A**	CEREBRORETINAL microangiopathywith calcifications and cystsis AND Leukoencephalopathywith vanishingwhite matter	Hypotonia- seizures- motor delay, Leukodystrophy EEG abnormality; Elevated urinary 3-hydroxybutyric acid; Hyperammonemia
M-5Y	YES	YES	POLR3B	NM_018082.5: c.3005 T > C	Hypomyelinatingleukodystrophy-8	Motor and languages delay, Leukodystrophy
F-4Y	YES	NO	MOCS1	NM_001075098.3:c.470G > A	Molybdenum cofactor deficiency of complementation group A	Delayed language development; Global developmental delay; Hearing impairment; Seizure
M-1Y	YES	YES	PNPO	**NM_018129.3: c.500 T > C**	Pyridoxamine 5-prime-phosphate oxidase deficiency	Seizure
M-4 M	YES	NO	GNPTAB	NM_024312.4:c.1547 A > T	Mucolipidosis II alpha/beta	Motor delay
M-6Y	YES	YES	NPC1	NM_000271.4:c.1553 + 6 T > C	Niemann-Pick disease C	Languages delay Ataxia-gait impairment, seizures

Novel mutations in bold, All mutations were homozygous, except those in Italics which were heterozygous

A total of 49 variants were identified: 25 (51%) were homozygous pathogenic or likely pathogenic variants, 19 (39%) were homozygous VUS, 1 patient (2%) had a heterozygous pathogenic variant, 3 patients (6%) had mitochondrial gene variants in a homoplasmic state, and 1 patient (2%) had a mitochondrial gene variant in a heteroplasmic state. Of the 49 variants identified, 14 (29%) were novel (Tables 3, 4).

### Consanguinity and genetic mutations

Except for 6 individuals, all patients with autosomal recessive diseases were from consanguineous families. These exceptions included patients with homozygous pathogenic variants in HEXA, MOCS1, EPG5, PET100, TTC19, and 1 with a homozygous variant of unclear significance in GCDH. Although these patients were not from consanguineous families, they shared a regional background. Patients with autosomal dominant mutations, such as the one with a pathogenic variant in the TPM2 gene, and those with mitochondrial mutations, including the patient with a variant in the MT-ND3 gene, were from non-consanguineous families (Tables 3 and 4).

### Autosomal recessive mutations

46% (25/54) had homozygous pathogenic or likely pathogenic variants, while 35% (19/54) had homozygous variants of uncertain significance. Notably, 14 of these mutations were novel (Tables 3 and 4).

### Autosomal dominant mutations

One patient had an autosomal dominant disorder, characterized by a heterozygous in-frame pathogenic mutation (class 1) in the TPM2 gene: TPM2, c.415_417 del p.(Glu139 del) (Table 3).

### Mitochondrial mutations

Four patients, three females and one male, were found to have known pathogenic mitochondrial mutations. The first patient harbored a 99.7% homoplasmic MT-ATP6, m.8563 A > T p.(Thr13Ser) mutation in the mitochondrial gene. The second patient, diagnosed with Leigh syndrome and NARP (neurogenic muscle weakness, ataxia, and retinitis pigmentosa), had a 91.1% heteroplasmic MT-ND3, m.10197G > A p.Ala47 Thr mutation. The third patient exhibited a 100% homoplasmic MT-ND3, m.10236 A > G p.Ile60 Val mutation. The last patient, a male, had a heteroplasmic MT-TS1, c.26 dupC mutation with a 37.5% heteroplasmic state (Table 5).

**Table 5:** Mitochondrial inheritance, All mutations are homoplasmic, except those in Italics, which are heteroplasmic,

Sex-age	Family history	Consanguinity	Genes	Variant	Diagnosis	Comments
F-8 y	No	Yes	MT-ATP6	VUS: NC_012920.1:m.8563 A > T (p.(Thr13Ser)	MT-ATP6 gene-associated disorder	Motor and languages delay, seizures
F-1Y	Yes	Yes	MT-ND3	VUS: NC_012920.1:m.10236 A > G (p.Ile60 Val)	Mitochondrial complex I deficiency nuclear type 5	Global developmental delay, seizures
F-2Y	Yes	No	MT-ND3	Pathogenic: NC_012920.1:m.10197G > A (p.Ala47 Thr)	Mitochondrial DNA (mt DNA)-associated Leigh syndrome and NARP (neurogenic muscle weakness, ataxia, and retinitis pigmentosa)	Hypotonia, gait impairment
M-4Y	Yes	Yes	*MT-TS1*	*Pathogenic: NC_012920.1:m.7471 dupC* (c.26 dupC)	Mitochondrial MT-TS1 gene-associated mitochondrial disorders	Global developmental delay, languages delay

### Impact of WES on diagnosis, treatment and patient’s outcome

**Fig. 3:**
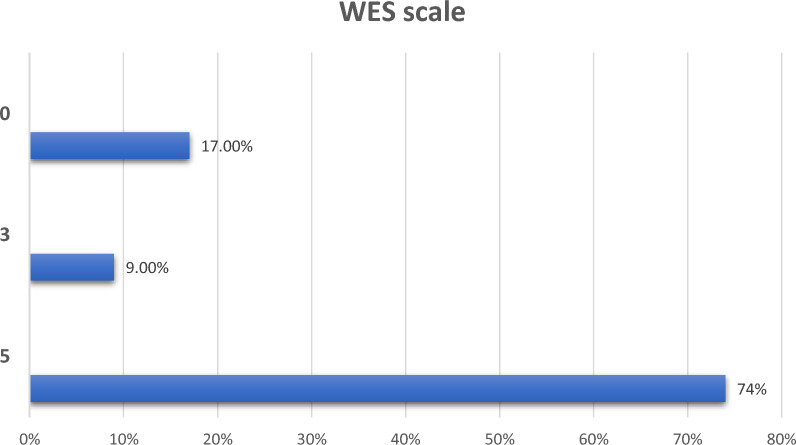
In the axis percentages of patients with retrospectively assigned impacts of their WES analysis

**Fig. 4:**
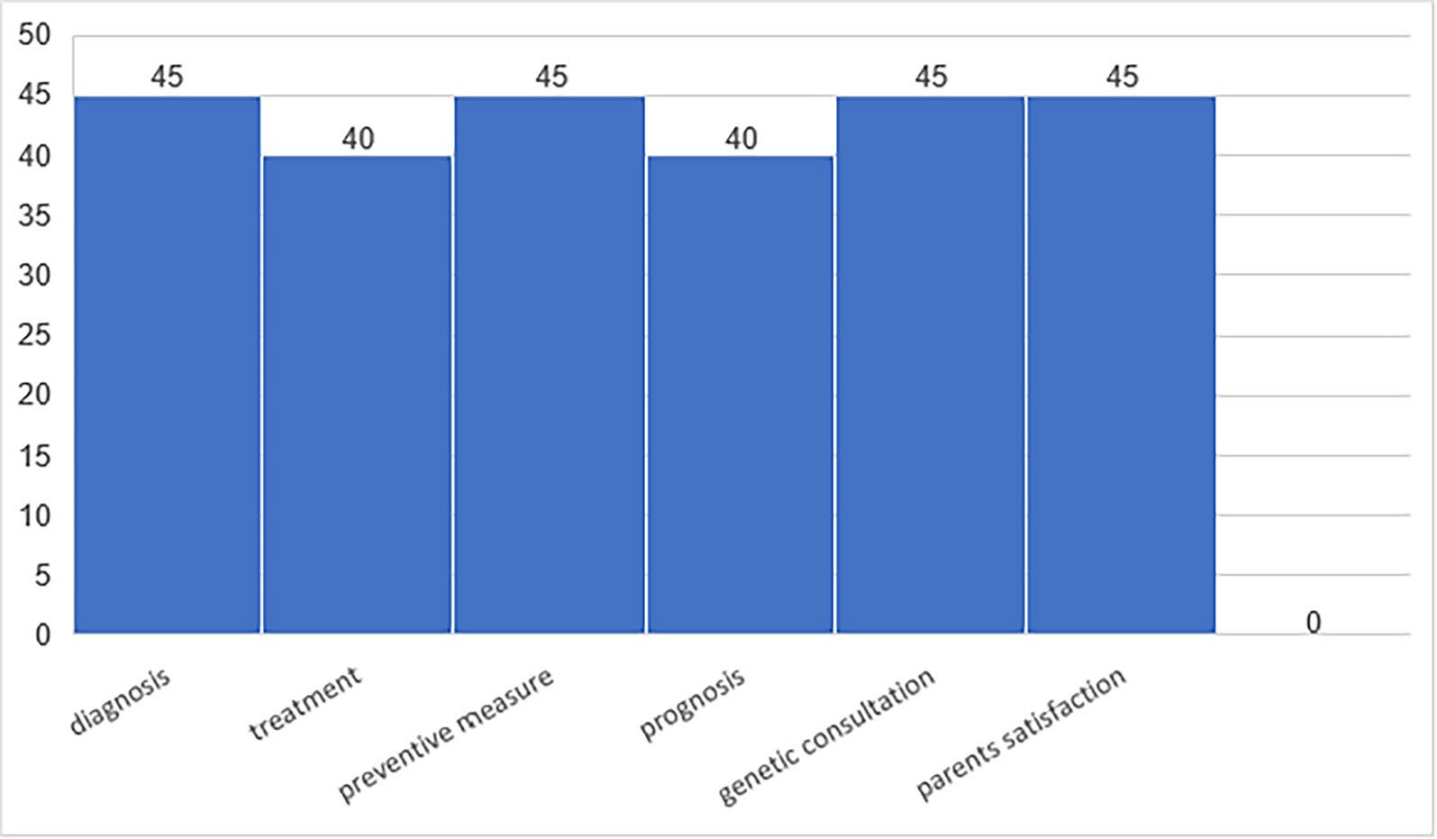
Effect of WES on changes in diagnosis, treatment plan, prognosis, genetic consultation, and parental satisfaction

In the diagnostic evaluation using WES, 40 patients (74%) achieved a maximal score of 5/5 on the WES scale, while 5 patients (9%) scored 3/5, and 9 patients (17%) scored 0/5. The median score for the entire sample was 5, with a range of 0–5 (Fig. 3 Notably, no cases scored 4/5. This absence may reflect the design of the scale, as it is structured to assign points cumulatively, and a 4-point combination was unlikely or absent in our cohort. WES led to a change in diagnosis for 45 patients (83%), altered the treatment plan for 40 patients (74%), modified the prognosis for 40 patients (74%), and prompted changes in preventive measures for 45 patients (83%) (Fig. 4). Additionally, genetic consultation was recommended based on WES results for 45 patients (83%), and 83% of the patients’ parents reported satisfaction with the WES test.

## Discussion

In this study, WES provided valuable clinical insights for the majority of patients, especially when other diagnostic tests were inconclusive. WES yielded clinically useful information in 83% (45/54) of patients and achieved a diagnostic rate of 52%, which is comparable to rates reported in similar studies from the UAE and Saudi Arabia (50%−53%) [1–4]. However, larger studies, such as Yang et al. [6], which analyzed 250 samples in the United States, reported a significantly lower diagnostic yield of 25% [6]. This discrepancy may reflect differences in the prevalence of autosomal recessive conditions and the impact of consanguinity in the respective populations. In our cohort, 81% (44/54) of patients had autosomal recessive conditions, compared to 29% in the U.S. study [6]. Additionally, 78% (42/54) of our patients had a family history of consanguinity, a notable increase compared to 69% reported in a Saudi Arabian study [1]. Consanguinity appears to be a critical factor in the diagnostic yield of WES, as evidenced by multiple studies. Al-Hedaithy et al. found that 83% of their cohort had a family history of consanguinity, and WES achieved a diagnostic yield of 56% in 53 patients with neuromuscular disorders. Importantly, their study highlighted that regions of homozygosity identified through a CGH array further supported the high prevalence of consanguinity, which was linked to autosomal recessive inheritance patterns [7]. Similarly, Turkdogan et al. reported a diagnostic yield of 39% for pathogenic or likely pathogenic variants, which increased to 61% when de novo variants linked to compatible phenotypes were included. In their study, 86% of patients were from consanguineous families, underscoring the role of consanguinity in identifying recessive and de novo variants [8].

Our study also highlighted the discovery of novel variants, with 31% (14/44) of the identified homozygous variants being previously unreported. This finding emphasizes the need for population-specific genetic databases, as novel mutations are often linked to consanguineous populations. Comparable findings were reported by Turkdogan et al., where 47% of the identified pathogenic variants were novel [8]. These data suggest that WES not only improves diagnostic accuracy but also contributes to expanding the understanding of rare genetic disorders in underrepresented populations.

Other studies have reported varying diagnostic yields depending on patient population and testing strategy. Triono et al. (2023), conducted in Indonesia, reported a diagnostic yield of 45% in children with suspected neurogenetic diseases at a tertiary referral hospital in Yogyakarta, with no mention of consanguinity [9]. Similarly, Kuperberg et al. (2015), found a diagnostic rate of 49% in pediatric neurological patients, highlighting the utility of WES for conditions like developmental delay and neuromuscular diseases [10]. Notably, Mitani et al. identified a causative molecular diagnosis in 75% of families, with multilocus pathogenic variations driven by runs of homozygosity observed in 29% of cases, further supporting the role of consanguinity in high-yield diagnostic outcomes [11].

Finally, the diagnostic yield of WES for neurological disorders remains consistently higher than in other conditions, Shickh et al.’s systematic review reported that neurological indications achieved higher diagnostic rates (22%−68%) than other indications. Furthermore, they highlighted the significant clinical utility of WES in guiding patient management, with changes to treatment, surveillance, and counseling observed in 4%−100% of cases [12].

WES proved particularly useful for patients with suspected mitochondrial disorders, in whom prior extensive work-ups had been inconclusive. Mutations in genes such as TTC19, SURF1, and PET100, which are associated with mitochondrial complex deficiency, as well as mutations in MT-ND3 linked to mitochondrial DNA-associated Leigh syndrome and NARP, were identified. Additionally, a mutation in MOCS1, associated with molybdenum cofactor deficiency of complementation group A, was found (Tables 3, 5). WES enabled a rapid diagnosis in these mitochondrial cases.

Furthermore, WES facilitated a prompt diagnosis in patients with treatable conditions such as primary coenzyme Q10 deficiency, BH4-deficient hyperphenylalaninemia type C, thiamine metabolism dysfunction syndrome, cerebral creatine deficiency syndrome, and pyridoxamine 5-prime-phosphate oxidase deficiency (Tables 3, 4). It also identified atypical presentations of rare diseases, including molybdenum cofactor deficiency and Galloway-Mowat syndrome. In addition, a patient with glutaric aciduria type 1 (GA-1) had normal tandem mass spectrometry (TMS) results, suggesting a low-excreter phenotype. This highlights the limitation of biochemical screening and further reinforces the role of WES in diagnosing neurometabolic disorders, especially when classical biomarkers are absent.

Physicians often conducted extensive and costly diagnostic workups before ordering WES, yet WES had a significant impact on clinical management in 83% of cases in our study. It led to changes in diagnosis (83%), treatment plans (74%), prognoses (74%), and preventive measures (83%), with genetic counseling recommended for 83% of patients. Parental satisfaction matched these outcomes (Fig. 3). Similar studies reported management changes in 33%−49% of cases, [13, 14] with molecular diagnoses enabling treatment adjustments, discontinuation of unnecessary interventions, and accurate recurrence risk estimates [15, 16].

In Syria, where consanguinity is common, WES has been valuable in reclassifying variants of uncertain significance. However, its broader application is hindered by limited access to sequencing technologies, financial constraints, and a shortage of trained personnel. Despite these challenges, the increasing use of WES, along with better understanding of allele frequencies, is expected to improve diagnostic outcomes. Ensuring the availability of trained personnel and adherence to guidelines from genetic societies, such as those from the American College of Medical Genetics, the Canadian College of Medical Genetics, and the European Society of Human Genetics is essential for accurate WES interpretation, ensuring consistent results across populations, and expanding its use in Syria and similar regions [17, 18].

## Conclusion

Our experience demonstrates the high diagnostic yield of WES and its significant impact on clinical outcomes. WES has led to changes in diagnosis, adjustments to treatment plans, modifications of prognoses, and the implementation of preventive measures. Moreover, genetic consultations were recommended based on WES results, and parents expressed satisfaction with the testing process, further supporting its use in undiagnosed neurometabolic diseases. This study advocates for the use of WES in pediatric neurometabolic disorders, particularly in cases with a family history of similar conditions and consanguinity. Future studies should address questions regarding cost-effectiveness, which will require prospective study designs.

## Data Availability

All data generated or analyzed during this study are included in this published article.

## Abbreviations

WES: Whole exome sequencing
VUS: Variants of uncertain significance
CDGs: Congenital disorders of glycosylation
